# Comparative analysis of the hemodynamic and metabolic effects of alternative fluid resuscitation strategies in critically ill patients with combined chest trauma and hemorrhagic shock: impact on oxygen delivery-consumption balance and coagulation homeostasis

**DOI:** 10.3389/fmed.2026.1891606

**Published:** 2026-10-14

**Authors:** Xianfei Ke, Zhi Hu, Tingzhong Wang, Yuejiao Wei, Xiaolin Hou, Qiang Song

**Affiliations:** 1 Department of Emergency, Renmin Hospital, Hubei University of Medicine, Shiyan, Hubei 442000, China; 2 Department of Cardiovascular Medicine, The First Affiliated Hospital of Xi ‘an Jiaotong University, Xi ‘an City, China; 3 Department of Cardiovascular Medicine, Sichuan Academy of Medical Sciences & Sichuan Provincial People's Hospital (Affiliated Hospital of University of Electronic Science and Technology of China), Chengdu City, Sichuan, China

**Keywords:** coagulation function, fluid resuscitation strategy, hemorrhagic shock, inflammatory factors, oxygen metabolism regulation, severe chest trauma

## Abstract

**Objective:**

This study aimed to investigate the effects of different fluid resuscitation strategies on clinical efficacy, oxygen metabolism, coagulation function, and inflammatory response in critically ill patients with chest trauma complicated by hemorrhagic shock, so as to provide evidence for optimizing emergency treatment strategies.

**Methods:**

A total of 107 patients with severe chest trauma and hemorrhagic shock who were admitted to our hospital between June 2023 and April 2025 were retrospectively enrolled. According to the type of treatment received, patients were divided into two groups. The control group (*n* = 55) received conventional fluid resuscitation, while the observation group (*n* = 52) received restrictive fluid resuscitation. The two groups were compared in terms of fluid resuscitation parameters, oxygen metabolism indicators [mixed venous oxygen saturation (SvO2), oxygen consumption (VO2), oxygen delivery (DO2)], coagulation indicators [activated partial thromboplastin time (APTT), prothrombin time (PT), hematocrit (HCT)], inflammatory indicators [interleukin-6 (IL-6), interleukin-10 (IL-10), tumor necrosis factor-α (TNF-α)], as well as mortality and complication rates during resuscitation.

**Results:**

Compared with the control group, the observation group had significantly lower fluid input volume (*P* < 0.05); there were no significant differences in early resuscitation time or colloid use between the two groups (*P* > 0.05). After resuscitation, SvO2, VO2, DO2, and HCT levels were all significantly increased compared with before resuscitation, while APTT, PT, IL-6, IL-10, and TNF-α levels were significantly decreased in both groups. The magnitude of changes in the observation group was significantly greater than in the control group (*P* < 0.05). During the resuscitation period, the survival rate of the observation group was significantly higher than that of the control group, while the incidence of ARDS, DIC, and MODS was significantly lower (*P* < 0.05).

**Conclusion:**

Compared with conventional fluid resuscitation, restrictive fluid resuscitation shows more significant clinical advantages. Its application in patients with severe chest trauma complicated by hemorrhagic shock can reduce unnecessary fluid overload, more effectively improve oxygen metabolism, correct coagulation dysfunction, suppress inflammatory responses, thereby increasing survival rates and reducing the risk of complications.

## Introduction

Severe chest trauma is one of the most common and life-threatening types of trauma emergencies, often accompanied by massive bleeding and severe respiratory and circulatory disorders (1). Statistics show that chest trauma accounts for approximately 10%–15% of all trauma cases, with a high incidence of hemorrhagic shock, which is a major cause of early mortality in such patients (2). Hemorrhagic shock caused by chest trauma not only leads to a sharp reduction in effective circulating blood volume but also results in insufficient tissue perfusion and oxygen supply depletion. If resuscitation is not carried out promptly or strategies are inappropriate, it may easily lead to multiple organ dysfunction and even death (3). Therefore, choosing an appropriate fluid resuscitation strategy is of great clinical significance for improving survival rates and prognosis. Traditional fluid resuscitation strategies focus on rapid blood volume replacement and hemodynamic stabilization, usually achieved through large-volume infusion of crystalloids or colloids (4). However, an increasing number of studies (5–7) have demonstrated that although conventional large-volume resuscitation can temporarily improve hemodynamic parameters, excessive fluid infusion often causes hemodilution, coagulation dysfunction, tissue edema, and exacerbated inflammatory responses, and may even trigger severe complications such as acute lung injury and abdominal compartment syndrome, thereby adversely affecting long-term outcomes.

Damage-control resuscitation principles, including avoidance of unnecessary crystalloid loading and the use of permissive hypotension before definitive hemostasis in appropriate patients, have been established for many years (8, 9). Within this framework, restrictive fluid resuscitation aims to provide the minimum effective volume required to preserve vital-organ perfusion while limiting dilutional coagulopathy and the risk of further bleeding. Relevant studies (10) suggest that restrictive fluid resuscitation may help alleviate coagulation dysfunction and inflammatory responses in patients with hemorrhagic shock, improve oxygen metabolism, and potentially enhance survival rates. However, the application of restrictive fluid resuscitation in patients with severe chest trauma and hemorrhagic shock remains controversial. Some research findings are inconsistent, and comprehensive evaluations involving multiple parameters such as oxygen metabolism, coagulation function, and inflammatory factors are still lacking. Based on this, the present study retrospectively analyzed 107 patients with severe chest trauma and hemorrhagic shock and compared less restrictive and restrictive early fluid resuscitation strategies across multiple clinically relevant domains, including oxygen metabolism, coagulation function, inflammatory responses, and early clinical outcomes. Rather than proposing a new resuscitation concept, this study aimed to provide population-specific real-world evidence regarding the application of restrictive resuscitation in patients with severe chest trauma and hemorrhagic shock.

## Materials and methods

### Study design and ethical statement

This study was a retrospective cohort study, including patients admitted to the Trauma Treatment Center of our hospital between June 2023 and April 2025 with severe chest trauma complicated by hemorrhagic shock. The study protocol was reviewed and approved by the Ethics Committee of our hospital (Ethics No.: ZZJJBY25-07LC). All case data were de-identified before analysis; written informed consent from participants was waived according to law, as this was a retrospective data analysis. The study complied with the principles of the Declaration of Helsinki and protected patient privacy and data security.

Inclusion criteria: (1) age ≥18 years, regardless of sex; (2) admission diagnosis of severe thoracic trauma, including multiple rib fractures/flail chest, chest wall collapse, extensive pulmonary contusion, massive hemothorax, and/or major thoracic vascular injury, complicated by hemorrhagic shock; (3) meeting clinical criteria of hemorrhagic shock (11): systolic blood pressure <90 mmHg at admission or signs of significant tissue hypoperfusion (cold clammy skin, altered consciousness, reduced urine output, etc.) and imaging or surgical confirmation of active bleeding source; (4) time from injury to admission <6 h, and fluid resuscitation performed due to bleeding/chest trauma; (5) Injury Severity Score (ISS) (12) > 16; (6) complete and reliable clinical data available for analysis.

Exclusion criteria: (1) history of severe congenital coagulopathy or long-term oral anticoagulant use that could not be restored to reference baseline; (2) combined severe craniocerebral injury [Glasgow Coma Scale (GCS) (13) ≤ 8 with primary death risk from brain injury]; (3) pregnancy, end-stage malignancy, or expected survival <1 month; (4) history of severe cardiac insufficiency (NYHA class Ⅲ–Ⅳ) (14) or severe hepatic/renal failure; (5) incomplete medical records with missing key indicators or lack of complete resuscitation records after transfer.

The 107 eligible patients were retrospectively classified into two groups according to the early resuscitation strategy documented in their medical records: a less restrictive fluid resuscitation group (control group, *n* = 55) and a restrictive fluid resuscitation group (observation group, *n* = 52). Grouping was based on the resuscitation strategy implemented by the admitting team during the initial trauma resuscitation phase and was retrospectively classified from the medical records. The group assignment therefore reflected the early resuscitation strategy before definitive hemostatic control and did not imply that the same group-specific fluid strategy was maintained throughout the postoperative period.

### Resuscitation strategies and treatment process

All patients were immediately managed through the trauma emergency protocol after admission, first ensuring airway patency and providing necessary respiratory support. Hemodynamic parameters were closely monitored, multiple venous access routes were established promptly, and invasive arterial monitoring was performed when necessary to ensure real-time dynamic monitoring of circulation and oxygenation. Initial management included hemostasis, fluid replacement, oxygen therapy, and maintenance of tissue perfusion, aiming to reverse hypoperfusion and hypoxia as quickly as possible, preserving essential function of the heart, brain, liver, kidneys, and other vital organs. During resuscitation, central venous pressure, arterial blood gas analysis, urine output, mental status, peripheral pulse quality (particularly the radial pulse), and peripheral perfusion were dynamically assessed to guide fluid management and evaluate resuscitation effectiveness. Hepatic and renal function were also monitored throughout the early resuscitation period.

In both groups, blood component therapy was not deferred until anemia was documented and was not contingent on completion of a fixed volume of intravenous fluid. In patients with ongoing or substantial hemorrhage and persistent hemodynamic instability, blood products were initiated during the initial trauma resuscitation, generally within 15 min of arrival in the Trauma Resuscitation Unit (TRU). For patients who remained hypotensive or showed persistent signs of tissue hypoperfusion during an initial crystalloid infusion of approximately 500 mL, packed red blood cells and plasma were started promptly; in patients with profound shock or obvious ongoing major bleeding, blood component therapy could be initiated concurrently with, or before completion of, the initial crystalloid infusion. Packed red blood cells were administered according to estimated blood loss, hemoglobin/hematocrit levels, and hemodynamic status, whereas plasma supplementation was guided by ongoing bleeding and coagulation abnormalities. The indications and general principles for blood component supplementation were the same in both groups; the actual amount administered was individualized according to the severity of blood loss, hemodynamic status, and coagulation abnormalities. The main between-group difference was therefore the fluid resuscitation strategy and corresponding blood pressure target rather than the transfusion strategy itself. The transfusion strategy recorded in this cohort was based on component therapy, principally packed red blood cells and plasma, and no fixed 1:1:1 transfusion protocol was prespecified as part of the study-group resuscitation strategy. The control group received conventional fluid resuscitation, following the principle of “rapid fluid replacement and early correction of hypotension.” Patients were administered crystalloids, primarily lactated Ringer's solution and 0.9% sodium chloride (normal saline), via rapid intravenous infusion immediately after admission. When colloid supplementation was considered necessary, 4% succinylated gelatin solution was used according to the patient's hemodynamic status and clinical assessment. The resuscitation goal was to restore and maintain systolic blood pressure above 90 mmHg as quickly as possible, and to maintain central venous pressure within normal range, thereby ensuring adequate organ perfusion. Blood components were supplemented according to the above criteria, with vasopressors used when necessary.

The observation group received restrictive fluid resuscitation, adopting a “limited fluid replacement and permissive hypotension” strategy to minimize the adverse effects of fluid overload on coagulation and tissue damage. Upon admission, two or more effective intravenous routes were established. Resuscitation was initiated with small volumes of crystalloids, primarily lactated Ringer's solution and 0.9% sodium chloride (normal saline), combined, when clinically indicated, with 4% succinylated gelatin solution. The crystalloid-to-colloid ratio was generally maintained at approximately 2:1 to 3:1, and the infusion rate was typically about 500 mL/h. A systolic blood pressure of approximately 70–75 mmHg was used as an initial reference range rather than an absolute target. The target was individualized according to the patient's age, pre-injury blood pressure history (including chronic hypertension), overall body habitus, mental status, radial pulse, urine output, and other signs of peripheral perfusion. If consciousness was preserved or improving, the radial pulse remained palpable, urine output was maintained at approximately ≥0.5 mL/kg/h, and no other signs of progressive hypoperfusion were present, the infusion rate was reduced after the reference pressure range was reached. Conversely, if mental status deteriorated, the radial pulse became weak or absent, urine output decreased, or other evidence of inadequate tissue perfusion persisted, fluid and blood-component resuscitation was intensified and a higher blood pressure was accepted as clinically necessary. During resuscitation, excessive crystalloid use was strictly limited. Blood components were supplemented according to the same clinical and laboratory criteria. The entire process emphasized maintaining essential organ perfusion with the minimal effective fluid volume, rather than pursuing complete normalization of blood pressure.

When indicated, blood component therapy had already been initiated during the initial trauma resuscitation and was continued as clinically required before, during, and after definitive hemostatic intervention; it was not delayed until hemodynamic stabilization or the postoperative period. After initial resuscitation success (blood pressure and circulation basically stabilized), patients with confirmed bleeding lesions requiring surgical intervention were promptly taken to surgery for trauma control and hemostasis. Postoperatively, fluid and transfusion management was individualized by the responsible surgical and critical care teams according to the patient's hemodynamic status, ongoing bleeding, organ perfusion, and laboratory findings. Postoperative management was not required to continue the original control- or observation-group resuscitation strategy and was not used to redefine group assignment.

### Observation indicators and detection methods

1. **Baseline data collection:** demographic information (age, sex), cause of injury (traffic accident, fall, crush injury, other), trauma mechanism (blunt or penetrating), wound status (open or closed), ISS score, and shock severity (mild, moderate, severe).
2. **Fluid resuscitation-related indicators:** total fluid input (total volume of crystalloids + colloids + transfusion from admission to end of early resuscitation, in mL), early resuscitation time (from admission/initiation of resuscitation to achievement of target hemodynamics), colloid use (mL). Postoperative fluid administration was not included in the group-specific early resuscitation exposure assessment.
3. **Oxygen metabolism indicators:** “Before resuscitation” (before initiation of resuscitation) and “After resuscitation” (defined as the time recorded in clinical notes at the end of early resuscitation), mixed venous oxygen saturation (SvO2), oxygen delivery (DO2), and oxygen consumption (VO2) were measured using an oxygen metabolism monitor.
4. **Coagulation function indicators:** peripheral venous blood (4 mL) was collected “Before resuscitation” and “After resuscitation.” An automated coagulation analyzer (Shenzhen Mindray Bio-Medical Electronics Co., Ltd., Model: C2000-A) was used to measure activated partial thromboplastin time (APTT), prothrombin time (PT), and hematocrit (HCT).
5. **Inflammatory factor indicators:** peripheral venous blood (4 mL) was collected “Before resuscitation” and “After resuscitation.” After centrifugation, the serum supernatant was separated. Serum interleukin-6 (IL-6), interleukin-10 (IL-10), and tumor necrosis factor-α (TNF-α) levels were measured using a double-antibody sandwich ELISA.
6. **Mortality and complications during resuscitation:** patient survival during resuscitation (defined as admission and initiation of resuscitation to the end of early resuscitation or first hemostasis control) and complication incidence, including acute respiratory distress syndrome (ARDS), disseminated intravascular coagulation (DIC), multiple organ dysfunction syndrome (MODS), and acute renal failure (ARF). These complications were identified from the contemporaneous diagnoses documented in the medical records by the treating clinical team during the defined resuscitation period; no *post hoc* reclassification of these diagnoses was performed for the present retrospective analysis.

### Statistical analysis

GraphPad Prism 8 was used for plotting; SPSS 22.0 was used for statistical analysis. Continuous variables were tested for normality using the Shapiro–Wilk test. Normally distributed variables were expressed as ($\bar{x}\pm s$) and compared using paired t-tests (within-group before/after comparisons) and independent-sample t-tests (between-group comparisons). Categorical variables were expressed as frequency (n) and percentage (%), and compared using *χ*^2^ test or Fisher's exact test. A two-sided test was used, with significance level *α*=0.05, and *P* < 0.05 was considered statistically significant.

## Results

### Comparison of baseline data between the two groups

There were no significant differences in baseline data such as gender, age, cause of injury, wound status, ISS score, and shock severity between the two groups (*P* > 0.05), indicating comparability. The mechanisms of injury included traffic accidents, falls, crush injuries, and other causes, while the thoracic injury spectrum included multiple rib fractures/flail chest, chest wall collapse, extensive pulmonary contusion, massive hemothorax, and major thoracic vascular injury. With respect to trauma mechanism, the cohort was predominantly composed of patients with blunt trauma (100/107, 93.46%), whereas penetrating trauma accounted for only 7/107 (6.54%); the distribution of blunt versus penetrating trauma did not differ significantly between the two groups (Fisher's exact test, *P* = 1.000). See Table 1.

**Table 1:** Comparison of baseline data between the two groups [$\bar{x}\pm s$, *n*(%)].

Variable	Control (*n* = 55)	Observation (*n* = 52)	*t/x^2^*	*P*
Gender	-	-	0.171	0.678
Male	37 (67.27%)	33 (63.46%)	-	-
Female	18 (32.73%)	19 (36.54%)	-	-
Age (years)	45.64 ± 10.59	44.91 ± 11.07	0.348	0.728
Cause of injury	-	-	0.085	0.769
Traffic accident injury	28 (50.91%)	25 (48.08%)	-	-
Fall injury	15 (27.27%)	14 (26.92%)	-	-
Crush injury	7 (12.73%)	9 (17.31%)	-	-
Other injury	5 (9.09%)	4 (7.69%)	-	-
Wound status	-	-	0.146	0.701
Open	21 (38.18%)	18 (34.62%)	-	-
Closed	34 (61.82%)	34 (65.38%)	-	-
ISS score (points)	28.46 ± 6.17	27.89 ± 6.05	0.482	0.630
Shock severity	-	-	0.082	0.774
Mild	14 (25.45%)	12 (23.08%)	-	-
Moderate	24 (43.64%)	22 (42.31%)	-	-
Severe	17 (30.91%)	18 (34.62%)	-	-
Trauma mechanism	-	-	-	1.000^a^
Blunt trauma	51 (92.73%)	49 (94.23%)	-	-
Penetrating trauma	4 (7.27%)	3 (5.77%)	-	-

a Fisher's exact test.

### Comparison of fluid resuscitation-related indicators between the two groups

Compared with the control group, the observation group had significantly lower total fluid input (2,062.57 ± 343.72 mL vs. 2,974.83 ± 518.96 mL, *P* < 0.001). No significant differences were observed in early resuscitation time (21.79 ± 6.81 min vs. 23.68 ± 7.65 min, *P* = 0.180) or the volume of 4% succinylated gelatin administered (822.63 ± 304.85 mL vs. 768.54 ± 245.37 mL, *P* = 0.313). See Table 2.

**Table 2:** Comparison of fluid resuscitation-related indicators between the two groups ($\bar{x}\pm s$).

Indicator	Control (*n* = 55)	Observation (*n* = 52)	*t*	*P*
Fluid input (mL)	2,974.83 ± 518.96	2,062.57 ± 343.72	10.656	<0.001
Early resuscitation time (min)	23.68 ± 7.65	21.79 ± 6.81	1.347	0.180
4% succinylated gelatin volume (mL)	768.54 ± 245.37	822.63 ± 304.85	1.013	0.313

### Comparison of oxygen metabolism indicators between the two groups

After resuscitation, the levels of SvO₂, VO₂, and DO₂ in both groups were higher than those before resuscitation, and the changes in the observation group were more significant than those in the control group (*P* < 0.05). See Figure 1.

**Figure 1:**
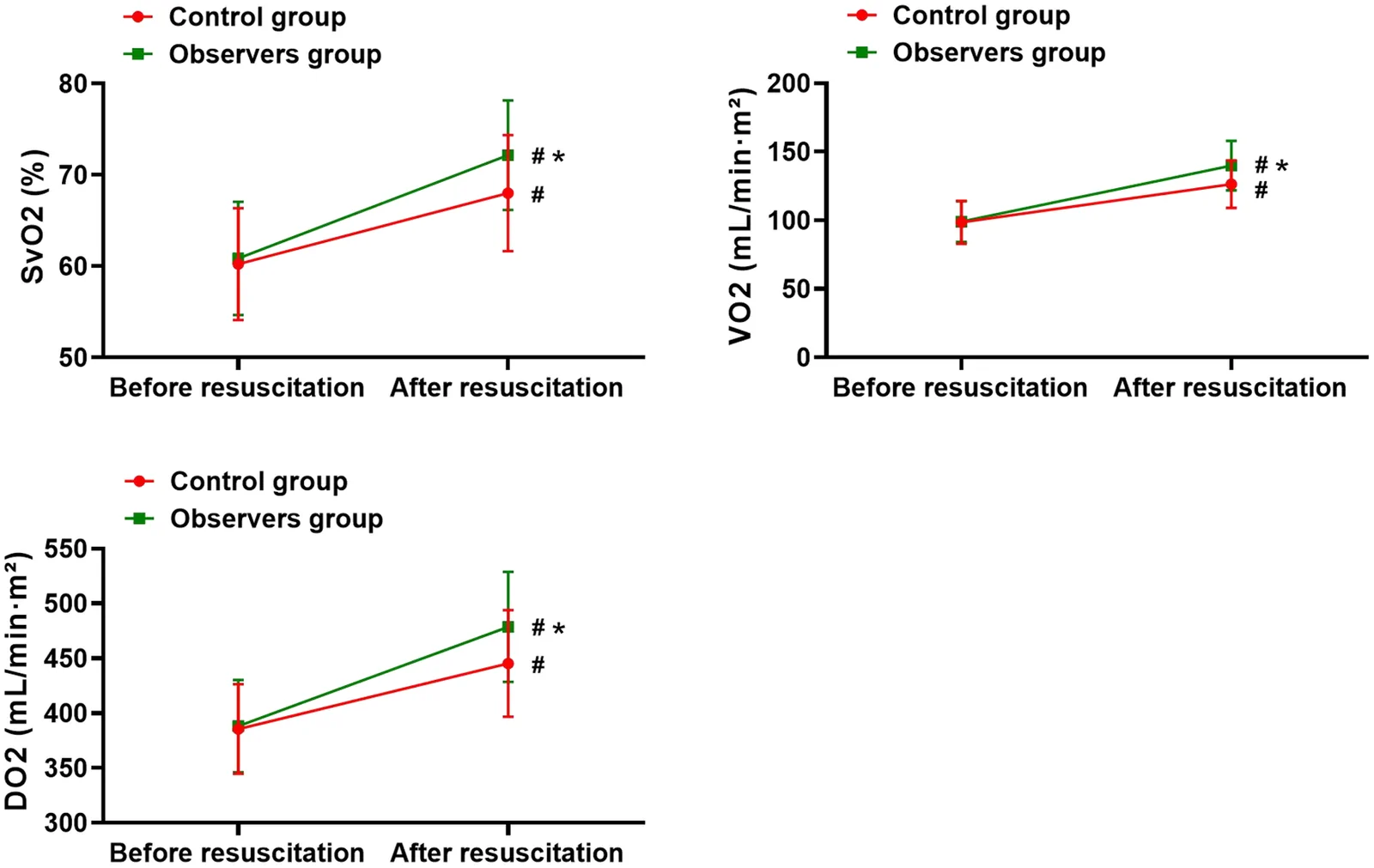
Comparison of oxygen metabolism indicators between the two groups ($\bar{x}\pm s$). Compared with before resuscitation in the same group, ^#^*P* < 0.05; compared between groups, *P < 0.05.

### Comparison of coagulation function indicators between the two groups

After resuscitation, the HCT levels in both groups were higher than those before resuscitation, while APTT and PT levels were lower than those before resuscitation, and the changes in the observation group were more significant than those in the control group (*P* < 0.05). See Figure 2.

**Figure 2:**
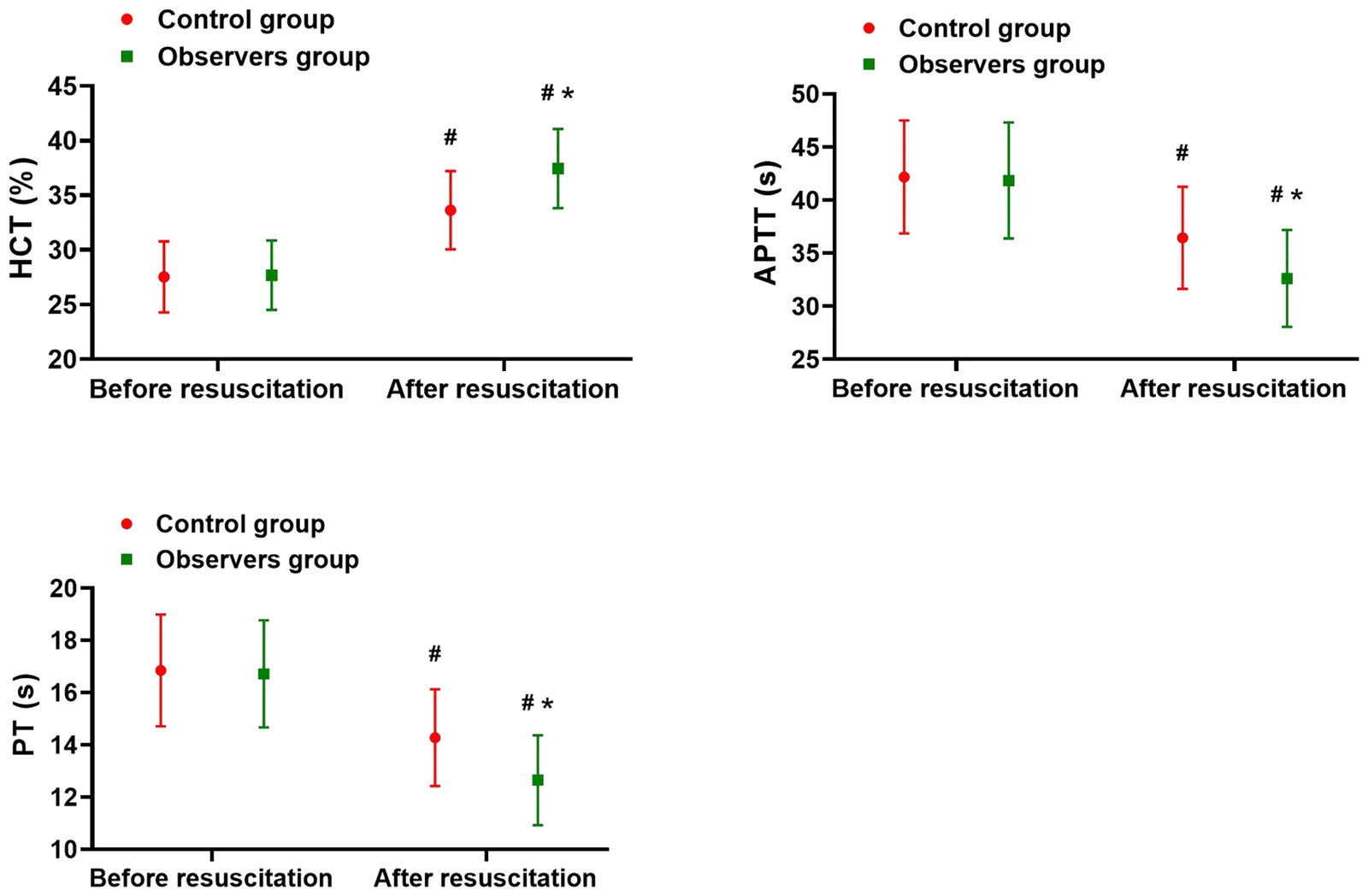
Comparison of coagulation function indicators between the two groups ($\bar{x}\pm s$). Compared with before resuscitation in the same group, ^#^*P* < 0.05; compared between groups, *P < 0.05.

### Comparison of inflammatory factor indicators between the two groups

After resuscitation, the levels of IL-6, IL-10, and TNF-α in both groups were lower than those before resuscitation, and the changes in the observation group were more significant than those in the control group (*P* < 0.05). See Figure 3.

**Figure 3:**
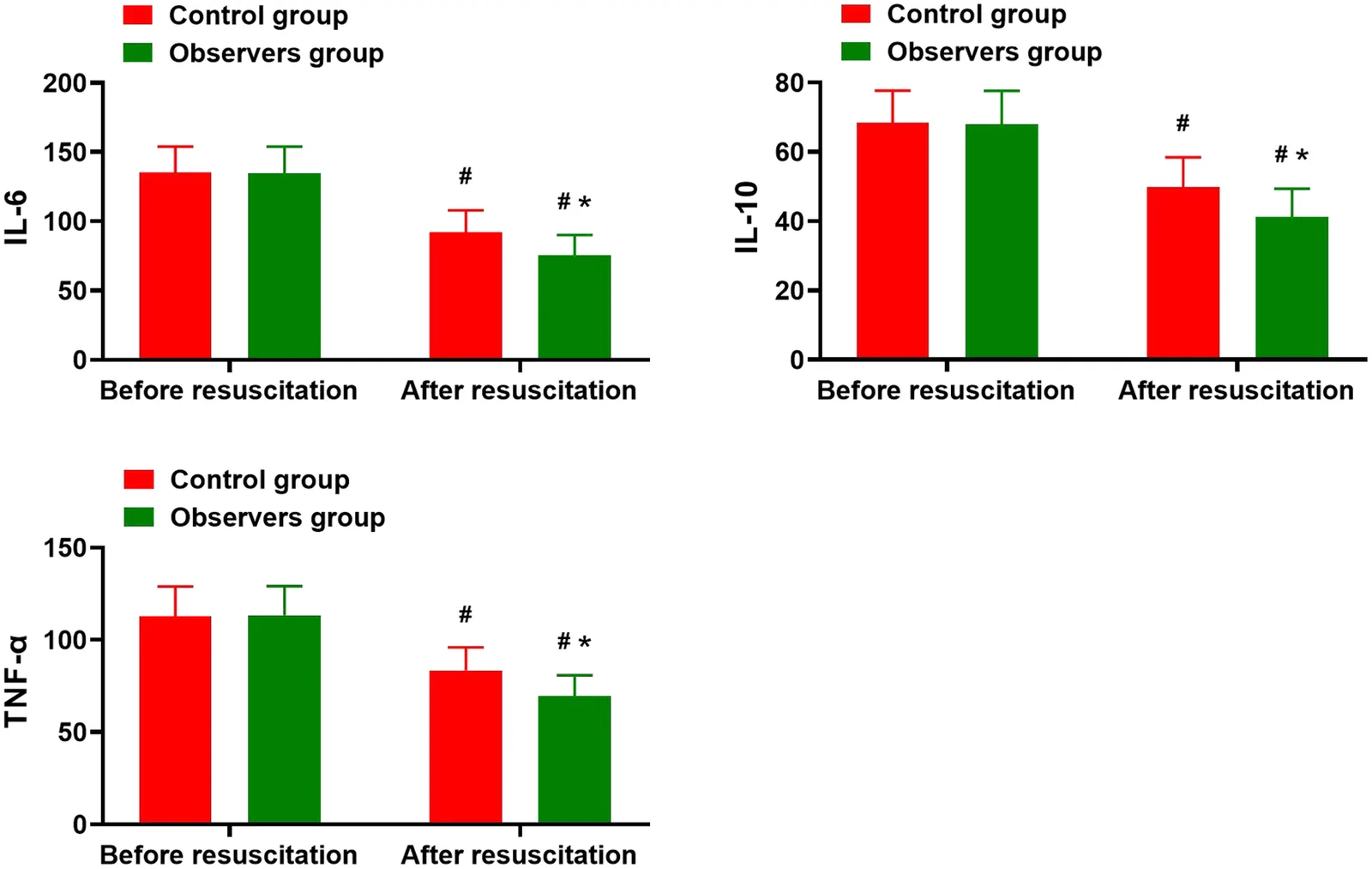
Comparison of inflammatory factor indicators between the two groups ($\bar{x}\pm s$, pg/mL). Compared with before resuscitation in the same group, ^#^*P* < 0.05; compared between groups, *P < 0.05.

### Comparison of mortality and incidence of complications during resuscitation between the two groups

During resuscitation, the survival rate was higher in the observation group than in the control group (50/52, 96.15% vs. 45/55, 81.82%; *P* = 0.018). The incidences of ARDS (15.38% vs. 34.55%; *P* = 0.022), DIC (5.77% vs. 20.00%; *P* = 0.029), and MODS (13.46% vs. 36.36%; *P* = 0.006) were lower in the observation group, whereas the incidence of ARF did not differ significantly between the groups (9.62% vs. 16.36%; *P* = 0.300). See Table 3.

**Table 3:** Comparison of mortality and incidence of complications during resuscitation between the two groups [*n*(%)].

Outcome	Control (*n* = 55)	Observation (*n* = 52)	*x^2^*	*P*
Survival rate	45 (81.82%)	50 (96.15%)	5.516	0.018
Complications	-	-	-	-
ARDS	19 (34.55%)	8 (15.38%)	5.201	0.022
DIC	11 (20.00%)	3 (5.77%)	4.759	0.029
MODS	20 (36.36%)	7 (13.46%)	7.431	0.006
ARF	9 (16.36%)	5 (9.62%)	1.070	0.300

## Discussion

Severe chest trauma combined with hemorrhagic shock is a highly challenging clinical problem in trauma emergency care. Patients often present within a short time with massive bleeding, impaired respiratory function, and insufficient circulatory perfusion. If resuscitation strategies are inappropriate, the condition can rapidly deteriorate, even leading to death (15, 16). Fluid resuscitation is a key step in treatment, but conventional large-volume crystalloid infusion, while rapidly restoring blood pressure, may exacerbate hemodilution, coagulation dysfunction, and tissue edema, ultimately resulting in organ failure (17, 18). In recent years, restrictive fluid resuscitation has attracted increasing attention. Its core concept lies in reducing excessive crystalloid infusion, maintaining low-level blood pressure, prioritizing correction of blood loss and restoration of microcirculation, thereby achieving the combined effects of “controlling bleeding, preserving perfusion, and reducing complications” (19). This study was based on this concept and compared the clinical outcomes of different resuscitation strategies in patients with severe chest trauma complicated by hemorrhagic shock.

The results of this study showed that the total fluid input in the observation group was significantly lower than that in the conventional resuscitation group, while no significant differences were found between the two groups regarding early resuscitation time and colloid use, suggesting that restrictive resuscitation did not delay the resuscitation process but rather reduced fluid load through more rational distribution. This finding is directionally consistent with that of Bickell et al. (20), who reported a benefit of delayed fluid resuscitation in hypotensive patients with penetrating torso injuries. However, the trauma populations are substantially different: 93.46% of patients in our cohort sustained blunt trauma, whereas the Bickell study focused on penetrating torso trauma. Blunt trauma may involve more diffuse tissue injury, pulmonary contusion, and occult or multifocal internal bleeding, whereas penetrating trauma more often produces a relatively focal injury tract and bleeding source. These differences can influence bleeding dynamics and the response to resuscitation. Therefore, the findings of Bickell et al. should be regarded as supporting the general principle of avoiding indiscriminate early crystalloid loading rather than as directly transferable evidence for patients with predominantly blunt thoracic trauma. In terms of oxygen metabolism, our findings revealed that SvO₂, VO₂, and DO₂ levels increased in both groups after resuscitation, but the increase was more pronounced in the observation group. These findings indicate that oxygen delivery improved after resuscitation in both groups, with a greater increase observed in the restrictive-resuscitation group. However, the higher DO₂ observed with restrictive resuscitation should not be explained solely by less hemodilution. Hemodilution may decrease measured hemoglobin and hematocrit concentrations without necessarily reducing the circulating red blood cell mass, while expansion of intravascular volume may increase preload and cardiac output and thereby partially or fully offset the reduction in arterial oxygen content. Accordingly, DO₂ reflects the combined effects of blood oxygen-carrying capacity and systemic blood flow rather than hemoglobin concentration alone. In the present study, the greater increase in DO₂ in the restrictive-resuscitation group may therefore reflect a more favorable overall balance between oxygen content, circulating volume, and perfusion, rather than a direct consequence of preserved hemoglobin or hematocrit. Because cardiac output, stroke volume, and circulating red blood cell mass were not independently quantified, the precise mechanism underlying the between-group difference in DO₂ cannot be determined from our data. Limiting excessive fluid administration may also reduce the aggravation of pulmonary interstitial edema and associated oxygenation impairment, thereby potentially supporting tissue oxygenation (21, 22). Hu et al. (23) also reported that restrictive resuscitation significantly reduced lactate levels and improved lactate clearance, which is consistent with the trend of improved oxygen metabolism indicators in our study.

Regarding coagulation function, the results indicated that patients in the restrictive resuscitation group had greater increases in HCT and more pronounced shortening of APTT and PT, suggesting better recovery of coagulation function compared with conventional resuscitation. Large-volume crystalloid infusion in traditional approaches dilutes coagulation factors and platelets, which is a key factor in exacerbating trauma-induced coagulopathy (TIC) (24). Conversely, restrictive resuscitation combined with early blood product supplementation can maintain coagulation factor concentrations and reduce dilutional coagulopathy. Holcomb et al., in a large-scale multicenter study, reported that early transfusion using a 1:1:1 ratio of plasma, red blood cells, and platelets significantly reduced mortality in trauma patients (25). Although the present study did not evaluate a fixed 1:1:1 transfusion protocol, these findings are broadly consistent with the principle of early, individualized blood component support within damage-control resuscitation. These findings should not be interpreted as suggesting that large-volume crystalloid resuscitation is an acceptable contemporary standard for patients with ongoing traumatic hemorrhage. Rather, they are consistent with established damage-control resuscitation principles: early blood component therapy when ongoing hemorrhage is suspected, avoidance of unnecessary crystalloid loading, and conservative blood-pressure and perfusion targets sufficient to reverse life-threatening hypoperfusion while definitive hemorrhage control is pursued. In the present retrospective cohort, the two groups reflected different degrees of fluid restriction and blood-pressure targeting within real-world early resuscitation practice, rather than a comparison between blood-based resuscitation and a crystalloid-only strategy. In terms of inflammatory response, this study showed that IL-6, TNF-α, and IL-10 levels decreased more markedly in the observation group than in the control group. Excessive crystalloid administration may contribute to endothelial glycocalyx injury, increased capillary permeability, and tissue edema (26); however, ARDS after severe thoracic trauma is multifactorial. Potential contributors include the primary pulmonary injury itself, transfusion-related acute lung injury (TRALI), secondary pulmonary infection or ventilator-associated pneumonia (VAP), and, in selected patients, hydrostatic pulmonary edema related to acute cardiac dysfunction. Therefore, although the restrictive-resuscitation group had a lower observed incidence of ARDS, this finding should not be interpreted as evidence that intravenous fluid overload was the primary cause of ARDS. Reduced fluid exposure may have limited aggravation of pulmonary edema or endothelial injury, but the present study cannot establish the specific mechanism responsible for the between-group difference. Our findings echo the animal study by Chi et al. (27), which confirmed in a hemorrhagic shock model that restrictive resuscitation effectively reduced plasma IL-6 levels and improved histological damage in multiple organs, suggesting that this strategy not only provides clinical benefits but also has a clear biological basis. In terms of final outcomes, our study revealed that the survival rate in the observation group was significantly higher than in the control group, while the incidence of complications (especially ARDS, DIC, and MODS) was significantly lower. These findings are consistent with trends reported in previous clinical studies (28–30). Taken together, the survival benefit of restrictive resuscitation derives from its multifaceted protective effects on oxygen metabolism, coagulation, and inflammation.

Of course, this study has certain limitations. First, as a single-center retrospective study with a limited sample size, selection bias may exist. Second, some key indicators, such as SvO₂, may have been substituted with ScvO₂ in certain patients, introducing measurement discrepancies. In addition, cardiac output, stroke volume, and circulating red blood cell mass were not independently quantified, limiting mechanistic interpretation of the observed between-group differences in DO₂. Furthermore, this study only evaluated the early effects of resuscitation and lacked assessment of long-term survival and quality of life. In addition, TEG/ROTEM data were not routinely available in this retrospective cohort; therefore, coagulation status was evaluated using conventional laboratory indices, which may not fully characterize the dynamic changes of trauma-induced coagulopathy. In addition, the respective volumes of lactated Ringer's solution and normal saline were not analyzed separately in this retrospective cohort; therefore, potential differences related to crystalloid composition, including chloride-associated acid-base effects and endothelial glycocalyx injury, could not be independently evaluated. Moreover, the retrospective records did not permit reliable etiologic adjudication of individual ARDS events, including differentiation among primary traumatic lung injury, TRALI, secondary pulmonary infection/VAP, and cardiogenic pulmonary edema; therefore, the observed between-group difference in ARDS incidence cannot be attributed specifically to fluid overload or to any single mechanism. Therefore, future multicenter, large-sample, prospective randomized controlled trials are needed, with the introduction of more refined monitoring methods (e.g., TEG/ROTEM, continuous lactate clearance monitoring) to further verify and refine this strategy. In summary, this study demonstrated that compared with conventional resuscitation, restrictive fluid resuscitation more effectively improves oxygen metabolism, coagulation function, and inflammatory status in patients with severe chest trauma and hemorrhagic shock, thereby reducing complication risks and improving survival during the resuscitation period. These results are not only consistent with the concept of damage control resuscitation but also provide strong evidence for clinical practice. However, during clinical application, individualized patient conditions and surgical hemostasis should be considered to avoid a “one-size-fits-all” approach and to maximize the clinical value of this strategy.

## Conclusion

The results of this study indicate that goal-directed fluid resuscitation strategies in patients with severe traumatic shock can significantly improve oxygen metabolism, optimize coagulation function, and reduce inflammatory factor expression, while reducing total fluid input. This enhances resuscitation efficacy and decreases complication incidence. Compared with the less restrictive resuscitation approach observed in this cohort, the restrictive strategy was associated with more favorable early clinical outcomes. These findings support established damage-control resuscitation principles emphasizing avoidance of unnecessary crystalloid exposure, preservation of adequate tissue perfusion, timely blood component therapy, and prompt definitive hemorrhage control. Given the retrospective single-center design, the results should be interpreted as supportive evidence rather than as establishing a new resuscitation standard, and further multicenter prospective studies are warranted.

## Data Availability

The original contributions presented in the study are included in the article/Supplementary Material, further inquiries can be directed to the corresponding author.
